# Correction: Honegger et al. Antimicrobial Efficacy of Five Wound Irrigation Solutions in the Biofilm Microenvironment In Vitro and Ex Vivo. *Antibiotics* 2025, *14*, 25

**DOI:** 10.3390/antibiotics14070710

**Published:** 2025-07-16

**Authors:** Anja L. Honegger, Tiziano A. Schweizer, Yvonne Achermann, Philipp P. Bosshard

**Affiliations:** 1 Department of Dermatology, University Hospital Zurich, University of Zurich, 8091 Zurich, Switzerland; anjalinn.honegger@uzh.ch (A.L.H.); tiziano.schweizer@bluewin.ch (T.A.S.); philipp.bosshard@usz.ch (P.P.B.); 2 Department of Cranio-Maxillo-Facial and Oral Surgery, University Hospital Zurich, University of Zurich, 8091 Zurich, Switzerland; 3 Internal Medicine, Hospital Zollikerberg, 8125 Zollikerberg, Switzerland

## Title Correction

There was an error in the original publication [1]. The title was misleading, as not all tested irrigation solutions are indicated to be used intraoperatively. Therefore, the title was changed to reflect more the overall anti-biofilm properties of tested irrigation solutions.

A correction has been made to the title:

Antimicrobial Efficacy of Five Wound Irrigation Solutions in the Biofilm Microenvironment In Vitro and Ex Vivo

## Text Corrections

There was an error in the original publication [1]. One of the five irrigation solutions, Octenilin, is explicitly not indicated for intraoperative use. However, although not stating it directly, our original article implied that the tested solution might be used for PJI treatment intraoperatively. Therefore, we performed the following: (1) focused overall more on the biofilm disruption in vitro instead of the PJI-treatment by slightly changing some wording and (2) added specific information that Octenilin is not indicated for intraoperative use. The specific changes are listed below:

1. A correction has been made to the Abstract, Background/Objectives Section:

This in vitro study investigated and compared the effect of five advanced wound irrigation solutions to reduce bacterial burden in biofilm microenvironment.

2. A correction has been made to the Abstract, Conclusions Section:

Advanced wound irrigation solutions have the potential to reduce bacterial burden in the biofilm microenvironment. However, their efficacy varies depending on bacterial species, growth state, and the composition of the irrigation solution. While Octenilin should be avoided for deep tissue irrigation due to its potential to cause tissue necrosis, the clinical benefit of wound irrigation solutions in infection prevention warrants further investigation in prospective clinical trials.

3. A correction has been made to the Introduction Section, Paragraph 3:

However, both chlorhexidine and non-diluted Betadine are not indicated for intraoperative irrigation due to concerns about toxicity, iodine allergies, and thyroid dysfunction [30]. Therefore, we chose to compare the efficacy of various novel non-alcohol-based wound irrigation solutions in reducing bacterial burden in the biofilm microenvironment in relevant laboratory models in order to explore a potential additional use beyond wound treatment.

4. A correction has been made to the Introduction Section, Paragraph 4:

Paragraph 4 has been deleted.

5. A correction has been made to the Results Section, Sub-Section 2.1, Paragraph 1:

Notably, Betaseptic, that is not indicated for wound irrigation but for skin disinfection, achieved complete eradication of *S. aureus*, *S. epidermidis*, and *C. acnes*, but failed to completely eradicate *E. coli.*

6. A correction has been made to the Discussion Section, Paragraph 1:

Since it is known that wound irrigation solutions exhibit rapid bactericidal activity against planktonic bacteria, we investigated whether these commercially available solutions were able to reduce the bacterial load in the PJI biofilm microenvironment in vitro.

7. A correction has been made to the Discussion Section, Paragraph 2:

However, it has to be noted that Octenilin is not indicated for intraoperative irrigation due to its potential to cause aseptic tissue necrosis due to the disruption of cell membrane integrity of mammalian cells when retained in tissue.

The authors state that the scientific conclusions are unaffected. This correction was approved by the Academic Editor. The original publication has also been updated.
