# Supplementary material for: Antimicrobial Efficacy of Five Wound Irrigation Solutions in the Biofilm Microenvironment In Vitro and Ex Vivo
Source: Antibiotics (Basel). 2025 Jan 3;14(1):25. doi: 10.3390/antibiotics14010025 (PMC11762658; doi:10.3390/antibiotics14010025)
Supplement: Supplementary file 1 [file antibiotics-14-00025-s001.zip › antibiotics-3355011-supplementary.pdf]

Supplementary Material for Comparative Assessment of Antimicrobial Efficacy of Surgical Irrigation Solutions in the Peri-prosthetic Joint Infection Microenvironment *in vitro* and *ex vivo*

Table S1. Dataset to Figure 1: Antimicrobial efficacy of the irrigation solutions on biofilms formed on titanium alloy (TAV) discs. (A) Log10 CFUs/ml reduction of the irrigation solutions on 6 days old S. aureus, S. epidermdis and E. coli, as well as 8 days old C. acnes biofilms formed on TAV discs. Results are depicted as relative reduction to Ringer's lactate solution (negative control) and are the mean (±SEM) of three independent experiments performed in duplicates. Betaseptic (red) is not a wound irrigation solution. (B) Heat-map indicating the relative log10 CFUs/ml reductions across all irrigation solutions and bacterial species. (C) Heat-map indicating the mean log10 CFUs/ml reduction of each irrigation solution across all bacteria. Statistical analysis in A and C were performed by Two-way ANOVA. Statistically significant reductions are indicated with asterisks: *, $p < 0.5$; **, $p < 0.1$; ****, $p < 0.0001$.

Figure 1A

Staphylococcus aureus	Log10 reduction					
	Preventia	Prontosan	Granudacyn	Actimaris	Octenilin	Betaseptic
	0.4	0	0.41	0.68	1.33	6.82
	1.48	1.93	1.17	0.51	1.68	7.31
	1.56	0.22	0.57	1.56	3.19	7.18

Staphylococcus epidermidis	Log10 reduction					
	Preventia	Prontosan	Granudacyn	Actimaris	Octenilin	Betaseptic
	1.14	-0.1	-0.07	0.01	0.49	6.67
	0.35	1.02	0.71	1.68	0.54	7.26
	0.4	-0.08	0.23	0.43	0.17	6.78

Escherichia coli	Log10 reduction					
	Preventia	Prontosan	Granudacyn	Actimaris	Octenilin	Betaseptic
	0.4	0	0.41	0.68	1.33	6.82
	1.48	1.93	1.17	0.51	1.68	7.31
	1.56	0.22	0.57	1.56	3.19	7.18

Cutibacterium acnes	Log10 reduction					
	Preventia	Prontosan	Granudacyn	Actimaris	Octenilin	Betaseptic
	1.58	1.84	0.51	2.93	3.02	7.4
	1.44	3.95	0.72	0.61	7.76	7.76
	1.93	0.84	0.95	2.09	7.05	7.05

Figure 1B

	Preventia	Prontosan	Granudacyn	Actimaris	Octenilin
S. aureus	1.15	0.64	0.72	0.92	2.06
S. epidermidis	0.63	0.28	0.29	0.7	0.4
E. coli	1.26	0.98	0.71	0.93	3.88
C. acnes	1.65	2.21	0.73	1.88	5.94

Figure 1C

Octenilin	3.07
Preventia	1.17
Actimaris	1.11
Prontosan	1.03
Granudacyn	0.61

Table S2. Dataset to Figure 2: Antimicrobial efficacy of the irrigation solutions in the S. aureus abscess communities (SAC) model. (A) Representative image of SACs grown in collagen. (B) Log10 CFUs/ml reduction of the irrigation solutions on 24h old SACs formed in plasma-supplemented collagen matrices. Results are depicted as relative reduction to Ringer's lactate solution (negative control) and are the mean (±SEM) of three independent experiments performed in duplicates. Betaseptic (red) is not a wound irrigation solution Statistically significant reductions are indicated with asterisks: ****, $p < 0.0001$.

Figure 2

Preventia	Prontosan	Granudacyn	Actimaris	Octenilin	Betaseptic
0.13	0.24	0.07	8.12	0.54	4.03
0.15	0.47	0.13	8.11	0.33	8.11
1.06	1.13	0.76	8.79	1.19	8.79
0.05	0.4	0	8.06	0.28	8.06
